# Should Kidney Transplantation be Offered to Patients with Body Mass Index >40?: PRO

**DOI:** 10.34067/KID.0000000624

**Published:** 2025-08-08

**Authors:** Amanda J. Vinson

**Affiliations:** Kidney Research Institute Nova Scotia and Division of Nephrology, Department of Medicine, Nova Scotia Health, Halifax, Canada

**Keywords:** cadaver organ transplantation, health equity, diversity, and inclusion, kidney transplantation, minority health and disparities, obesity, transplantation

More than a billion adults worldwide are overweight or obese. In most developed countries, the prevalence of obesity among patients with ESKD on dialysis has increased, and, as a consequence, a greater number of obese patients on dialysis are being referred for kidney transplantation. Although recipient obesity may contribute to a higher risk of cardiovascular, metabolic, and surgical complications after kidney transplant,^1^ transplantation of obese individuals still confers a considerable survival advantage compared with remaining on dialysis.^2^ One study demonstrated that for patients with class 3 obesity (body mass index [BMI] >40 kg/m^2^), relative to remaining on dialysis, transplantation with a standard criteria donor reduced mortality risk by nearly 50% 1 year after transplant and by nearly 70% after receipt of a living donor kidney transplant.^2^ Although patients living with obesity experience an increased risk early after transplant, they still derive a net survival advantage with transplantation approximately 8 months following standard criteria donor and approximately 5 months after living donor transplant, relative to remaining on the transplant waitlist.^2^ Importantly, although obesity has been associated with a higher risk of cardiovascular disease, it has been shown that screening for and excluding patients with preexisting cardiovascular disease results in no differences in post-transplant survival between recipients with and without obesity.^3^ If the aim of restricting access to transplant for patients with an elevated BMI is to improve individual outcomes (acknowledging the higher risk of early post-transplant complications for obese versus normal-weight recipients), a more holistic view of overall outcomes indicates this is counterproductive; the counterfactual scenario of patients with obesity remaining on dialysis is of far greater risk.

Not only do patients living with ESKD and obesity garner benefit from kidney transplantation, but it is important to recognize the many limitations of BMI as a measure of obesity, a further argument that BMI is not a reliable metric to assess and ultimately restrict transplant eligibility. Reliance on BMI to define obesity can lead to misclassification of obesity status because it fails to distinguish between fat, muscle, and bone mass.^4^ In addition, BMI does not take into consideration the distribution of body fat, an important factor in metabolic disease risk. For example, android fat distribution (*e.g*., abdominal, central, visceral, or upper body fat distribution) is associated with an increased risk of cardiovascular disease and type 2 diabetes, whereas gynoid fat distribution (*e.g*. larger hip and thigh circumferences) seemingly is not. Likewise, the presence of visceral and ectopic fat has important implications for metabolic and adiposity-related risk; neither is identified by BMI.^5^ For example, visceral (but not subcutaneous) fat accumulation is associated with the increased secretion of free fatty acids, hyperinsulinemia, insulin resistance, hypertension, and dyslipidemia, with corresponding metabolic and cardiovascular consequences. Although there is significant overlap between the phenotypes of obesity, which type of obesity (*e.g*., visceral, ectopic, android, gynoid, overall) best correlates with adverse outcomes after kidney transplantation is unknown, though notably, BMI is not capable of differentiating between these specific obesity profiles. At least one prior study in kidney transplant recipients showed that when adjusting for waist circumference (which was significantly associated with increased post-transplant mortality), a higher BMI was paradoxically associated with *lower* post-transplant mortality; the same has been shown for elderly patients and those on dialysis.^6^ This may reflect a higher muscle mass and nonvisceral adiposity in patients with waist circumference–adjusted elevations in BMI.

Importantly, compounding the inability of BMI to characterize body fat distribution, the predictive capacity of BMI differs between patient subgroups, with considerable variation in BMI-defined risk by sex, age, cause of ESKD, and race.^7^ BMI does not reflect the normal physiologic changes that occur with aging. Body fat generally increases with advancing age while muscle mass decreases, with little corresponding changes in height or weight, nor, therefore, BMI.^4^ At the same BMI, female patients typically have 10% higher total adiposity than male patients, whereas male patients have higher visceral adipose tissue—a stronger predictor of metabolic consequences than total body fat. As a result, female patients are relatively protected from obesity-associated consequences; premenopausal women with obesity are at lower risk of diabetes and other metabolic complications than obese men.^8^ Importantly, patients with polycystic kidney disease may also have an inflation of their BMI on account of bulky kidneys, which does not correlate with true adiposity. In one study, nephrectomy of large polycystic kidneys reclassified 28.8% of patients into a lower BMI-defined obesity category.^9^ Finally, the ability of BMI to ascertain adiposity and obesity status varies considerably by race. In fact, experts have suggested a broad recalibration of BMI on the basis of race and sex-based risk,^7^ and country-specific cut points have already been developed for Asian subpopulations (*e.g*., China), for which BMI cut points of 24 kg/m^2^ for overweight and 28 kg/m^2^ for obesity are recommended. Similarly, recalibration of BMI has been proposed for Black populations, given differences in anthropometrics and body composition (*e.g*., bone mineral density, subcutaneous and visceral fat content, muscle mass) that may predispose to overestimation of obesity by BMI criteria. Correspondingly, a recent study demonstrated that an elevated BMI differentially predicts long-term risk in Black and White kidney transplant recipients; BMI-defined obesity was significantly associated with a greater risk of death-censored and all-cause graft loss in White recipients, but an attenuated risk of death-censored graft loss in Black recipients and no risk of all-cause graft loss.^10^ If the aim of restricting access to transplant for patients with an elevated BMI is meant to identify a subpopulation at greater risk of adverse post-transplant outcomes to maximize organ longevity from a societal and utilitarian perspective, then BMI—fraught with measurement error—is likely not the correct metric to define this risk.

Finally, even if the above concerns are overlooked, an additional consideration is that the institution of uniform BMI thresholds to define transplant eligibility has the potential to further exacerbate existing race- and sex-based inequities in access to transplant. Women and Black individuals are disproportionately affected by obesity^8^ and, therefore, may be overly disadvantaged by restrictive BMI criteria. This is of particular concern given there is minimal appreciable long-term risk after kidney transplant in Black individuals with BMI-defined obesity,^10^ and although not studied in the transplant setting, the implications of obesity differ by sex with fewer obesity-related metabolic consequences in female versus male patients in the general population. Therefore, not only are these populations more likely to be deemed ineligible for transplant on account of BMI criteria, but they are also less likely to be negatively affected by BMI-defined obesity. The same may be said of patients with ESKD on account of large polycystic kidneys. Therefore, if the goal of instituting a uniform BMI threshold to define transplant eligibility is to ensure an objective and unbiased approach to transplant assessment in the pursuit of equity, restricting access on the basis of a metric that will disproportionately disadvantage certain subpopulations (even when these groups may experience less obesity-related consequences than the population at large) is likely inappropriate; BMI thresholds to define transplant eligibility may paradoxically perpetuate existing disparities in access.

Given the limited pool of available donor kidneys, transplant programs are tasked with the responsibility of ensuring that kidneys are ethically and equitably allocated to recipients with a reasonable expectancy of survival after transplant. Importantly, however, the aim of maximizing the utility of organ allocation must be balanced against the principles of justice and fairness; even if data show consistently worse transplant outcomes for patients living with BMI-defined obesity (which is in itself controversial), patients must be assessed individually rather than by group membership. For example, it is well established that age, race, and sex all influence post-transplant outcomes, yet it would be inappropriate to limit access to transplant on the basis of these demographics (indiscriminately for the case of age). Likewise, the wide sweeping incorporation of a uniform BMI cutoff to define transplant eligibility may also be considered to oppose the principles of justice and respect for persons, particularly in light of the clear survival benefit with transplantation for otherwise eligible individuals living with obesity.

In conclusion, although the rationale to restrict access to transplant for those with a BMI >40 kg/m^2^ may theoretically make sense (*e.g*., minimize the known perioperative risk for such patients and improve utility at a societal level by avoiding transplant in patients with lower anticipated benefit), at a deeper level, this is likely counterproductive and short sighted. The survival for patients living with obesity is significantly improved by transplant versus remaining on dialysis, BMI as a measure of obesity is fraught with error, with a tendency to overclassify obesity in certain patient subgroups, BMI-defined obesity is not ubiquitously associated with increased risk with important variations between populations, and finally, the uniform institution of BMI thresholds to define transplant eligibility is likely to paradoxically increase existing race and sex-based inequities in access to transplant. For these reasons, while transplant health care providers can certainly encourage and help facilitate weight loss strategies for patients before transplantation, overtly restricting access to transplant for patients with ESKD living with obesity is likely inappropriate.

## Supplementary Material

SUPPLEMENTARY MATERIAL
