# Supplementary material for: Should Kidney Transplantation be Offered to Patients with Body Mass Index >40?: PRO
Source: Kidney360. 2025 Aug 8;6(12):2061–3. doi: 10.34067/KID.0000000624 (PMC12708391; doi:10.34067/KID.0000000624)
Supplement: SUPPLEMENTARY MATERIAL [file kidney360-6-2061-s001.pdf]

ASN American Society of Nephrology
JASN Journal of the American Society of Nephrology
KIDNEY360 Accessing Our World From Every Angle
CJASN Clinical Journal of the American Society of Nephrology

ASN Journal Disclosure Form

As per ASN journal policy, I have disclosed any financial relationships or commitments I have held in the past 36 months as included below. I have listed my Current Employer below to indicate there is a relationship requiring disclosure. If no relationship exists, my Current Employer is not listed.

A. Vinson reports the following:
Consultancy: Paladin Labs Inc., and Takeda Pharmaceuticals; Research Funding: Paladin Labs Inc.; Advisory or Leadership Role: Paladin Labs Inc., and Takeda Pharmaceuticals; and Other Interests or Relationships: Associate Editor for Kidney 360.

I understand that the information above will be published within the journal article, if accepted, and that failure to comply and/or to accurately and completely report the potential financial conflicts of interest could lead to the following: 1) Prior to publication, article rejection, or 2) Post-publication, sanctions ranging from, but not limited to, issuing a correction, reporting the inaccurate information to the authors' institution, banning authors from submitting work to ASN journals for varying lengths of time, and/or retraction of the published work.

Name: Amanda Jean Vinson
Manuscript ID: K360-2024-000840
Manuscript Title: DEBATE: Should Kidney Transplantation Be Accessible to Patients Living With Morbid Obesity (BMI >40 kg/m2) -PRO Position
Date of Completion: October 7, 2024
Disclosure Updated Date: October 7, 2024
